# Morphologic Characteristics of Choroid in the Major Choroidal Thickening Diseases, Studied by Optical Coherence Tomography

**DOI:** 10.1371/journal.pone.0147139

**Published:** 2016-01-14

**Authors:** Hoyoung Lee, Kunho Bae, Se Woong Kang, Se Joon Woo, Na-Kyung Ryoo, Sang Jin Kim, Gyule Han

**Affiliations:** 1 Department of Ophthalmology, Samsung Medical Center, Sungkyunkwan University School of Medicine, Seoul, Korea; 2 Department of Ophthalmology, Seoul National University Bundang Hospital, Seoul National University College of Medicine, Seongnam, Korea; Tohoku University, JAPAN

## Abstract

We investigated morphologic features of choroid in the choroidal thickening diseases, including central serous chorioretinopathy (CSC), polypoidal choroidal vasculopathy (PCV), and Vogt-Koyanagi-Harada disease (VKH), by a novel tomographic classification system of the choroid. This cross-sectional study involved 30 patients with active CSC, 30 patients with active PCV, and 27 patients with active VKH, and 30 normal controls. Utilizing enhanced depth imaging optical coherence tomography, we classified the morphology of the choroid into five categories: 1) Standard (S), 2) Dilated outer layer and Attenuated inner layer (DA), 3) Darkened (D), 4) Marbled (M), and 5) Pauci-Vascular (PV) types. Additional tomographic characteristics of the choroid such as choroidal vascular dilation, convolution, scleral invisibility, and choroidal hyper- or hypo-thickening were identified as well. The distribution of five choroidal tomographic morphology and additional tomographic characteristics in each group were analyzed. The DA type was observed in the CSC group more frequently than in the normal control group (53.3% vs 3.3%, *P* < 0.001). Additional tomographic characteristics, such as choroidal vascular dilation (76.7%), and choroidal hyper-thickening (36.7%), were more prevalent in the CSC group than in the control group. The PCV group showed higher prevalence of DA type (33.3% vs. 3.3%, *P =* 0.006) than the control group. The VKH group showed a significantly higher frequency of the D type (63.0%), convolution (40.7%), and scleral invisibility (70.4%) than controls (0% for all three findings). In conclusion, CSC and PCV shared common morphologic characteristics of choroid, including dilated outer vascular layer and focally attenuated innermost layer. Dense hypo-reflectivity and convolution of choroid were the specific tomographic markers for acute VKH. A new tomographic classification system of choroid may provide discrimination ability and insight into major pachychoroidopathies.

## Introduction

The recent advent of tomographic imaging techniques of deeper layers, such as enhanced depth imaging optical coherence tomography (EDI-OCT) or swept source OCT, has enabled in-vivo cross-sectional imaging of the choroid [1–6]. Understanding of such tomography is essential for understanding the structure of the normal choroid and for identifying structural changes in diseases that affect the choroid. Although their exact pathophysiology is not yet fully understood, central serous chorioretinopathy (CSC), polypoidal choroidal vasculopathy (PCV), and Vogt-Koyanagi-Harada disease (VKH) commonly show choroidal thickening [6–12]. They also share a common pathological feature of retinal pigment epithelium (RPE) and choroid dysfunction [12–16]. To our knowledge, no report exists which qualitatively assesses the tomographic morphologic differences between these diseases that share the common feature of choroidal thickening.

The purpose of this study is to identify the morphologic characteristics of the major pachychoroidopathies (CSC, PCV, and VKH) by applying a novel tomographic classification system of the choroid.

## Methods

We reviewed the Samsung Seoul Hospital and Bundang Seoul National University Hospital Department of Ophthalmology medical records for the period between January 1, 2010 and July 31, 2014 to identify cases of patients in active stages of CSC, PCV, or VKH (Datasets are included in S1 Appendix). Thus, the cases reviewed included both initial attack of the disease and recurrent cases. All cases had to have available diagnostic imaging modalities including enhanced depth imaging OCT. Latest consecutive cases were assembled retrospectively, that fulfill all requirements to engage 30 cases for each disease group. Only 27 cases could be secured for the VKH group, despite searching up to the 2010 database. The study was approved by the institutional review board, and the research adhered to the tenets set forth in the Declaration of Helsinki. Patient records were anonymized and de-identified prior to analysis.

Active CSC was defined as a condition showing serous elevation in the macular region under fundoscopic examination with subretinal leakage on fluorescein angiography (FA), choroidal hyperpermeability on late-phase Indocyanine green angiography, and detachment of the neurosensory retina confirmed on OCT [13, 14]. Diagnosis of active PCV was based on Indocyanine green angiographic findings, including a branching vascular network that terminated in polypoidal vascular abnormality. Only eyes with subretinal or sub-RPE fluid on OCT were included. Active VKH was diagnosed based on the criteria reported by an international committee on nomenclature [15]. In bilateral cases, eyes were randomized for inclusion of one eye.

Eyes with a myopic refractive error of ≥ -6.0 diopters, with evidence of other retinal or choroidal diseases, or with a history of photodynamic therapy were excluded, in addition to eyes which had received intravitreal injection within the three month period prior to diagnosis. In PCV, OCT grading was difficult in 7 out of 37 consecutive cases due to relatively extensive submacular hemorrhage and was excluded. In other diseases, grading with OCT classification was available in all cases with enhanced depth imaging-OCT imaging in the active phase.

In total, 30 eyes with CSC, 30 eyes with PCV, and 27 eyes with VKH were included in the study. All patients had undergone FA, and Indocyanine green angiography (Spectralis® HRA + OCT, Heidelberg Engineering Inc.) was performed in all patients with CSC and PCV. The normal control group consisted of 30 consecutively collected normal fellow eyes of unilateral epiretinal membrane patients who visited the clinic during the same period.

Both horizontal and vertical sections of EDI-OCT (Spectralis® HRA + OCT, Heidelberg Engineering Inc.) images were obtained in all eyes from the normal control and disease groups. We masked the retinal lesion revealing the disease by eliminating the image above the RPE to avoid bias in choroidal classification. An unmasked investigator (GLH) randomly assigned the order of each image. Three independent, blinded observers (SWK, SJW, and HYL) then classified the choroid into one of the five types.

**Fig 1:**
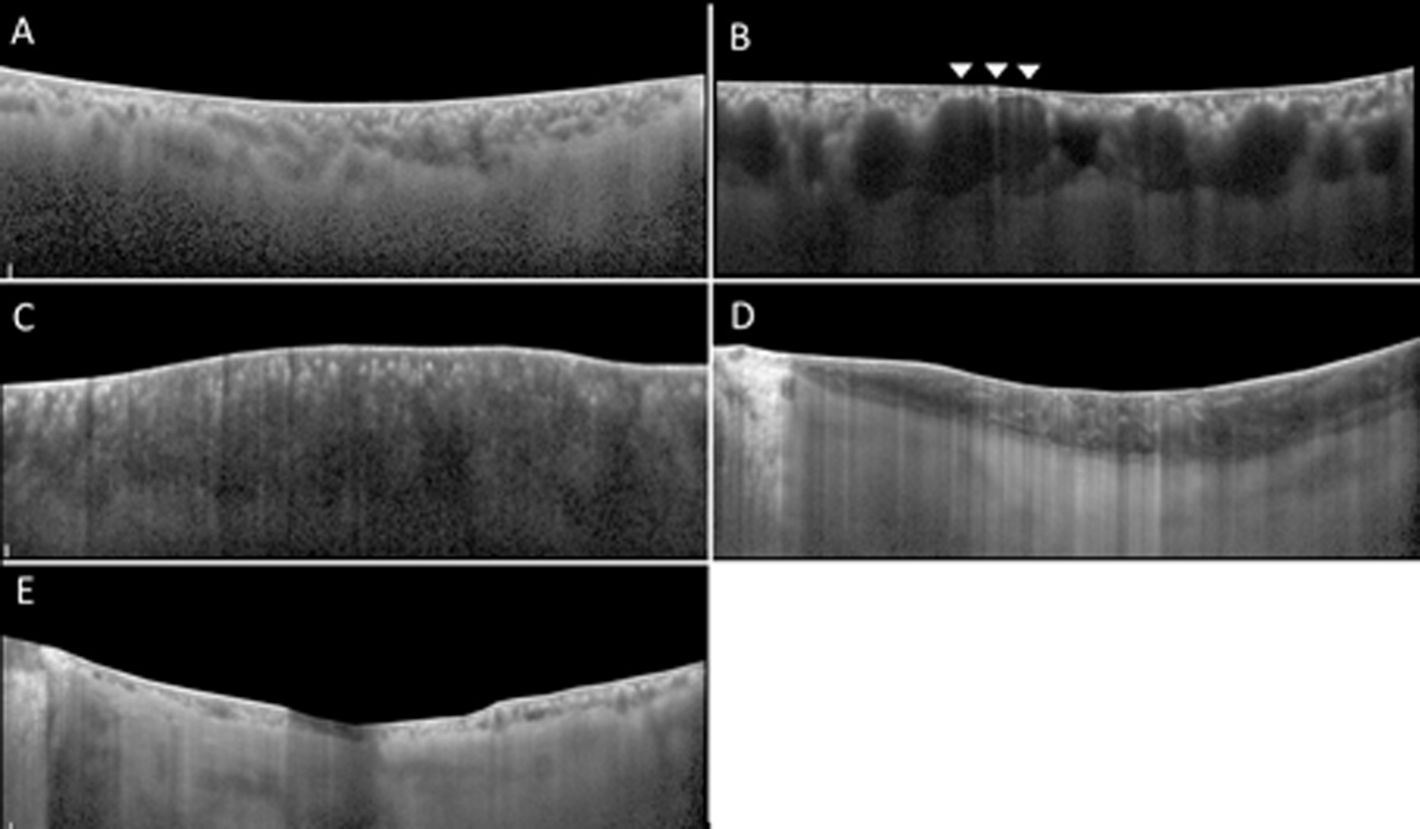
Representative images of the choroid. These demonstrate 5 types of tomographic classification in current study, after elimination of retinal image above retinal pigment epithelium. Standard (S) type (Fig 1A) shows clear visualization of the inner choriocapillaris layer, medium choroidal vessel layer and large choroidal vessel layer. The choroidal vessels are irregularly distributed among the choroidal layer and its lumen heterogeneous. The chorio-scleral border is relatively distinct and well-defined. Dilated outer layer and Attenuated inner layer (DA) type (Fig 1B) shows dilation of large choroidal vessel accompanied with the invisibility of innermost layer in the focal area of choroid (arrowheads). Darkened (D) type (Fig 1C) shows diffuse homogenous hyporeflectivity and, markedly decreased visibility of the outer choroidal layer. Marbled (M) type (Fig 1D) shows slightly dim choroidal reflectivity with obscured vessel margins. Multiple amorphous hyperreflective materials are dispersed within layers. In Pauci-Vascular (PV) type (Fig 1E), there are a lack of large choroidal vessels with choroidal thickness less than 100 um in the fovea and extrafoveal areas.

The five tomographic classifications of choroid morphology were defined as below (Fig 1):

- Standard (S) type was defined by clear visibility of the choriocapillaris/medium choroidal vessel layer and large choroidal vessel layer [17], and large choroidal vessel layer with irregular distribution of the heterogeneous choroidal vessels among the choroidal layers, and a relatively distinct and well-defined chorio-scleral border (Fig 1A).
- Dilated outer layer and Attenuated inner layer (DA) type was defined by attenuation of the inner vascular layer just above the dilated large choroidal vessel in the focal area of choroid (Fig 1B). Attenuation of the inner vascular layer was defined as invisibility or attenuation of hyperreflective spots immediately below the Bruch’s membrane. Fong et al., reported such hyperreflective spots as the cross section of arterioles and venules lying in parallel [8].
- Darkened (D) type was defined by diffuse, homogenous, hypo-reflectivity and markedly decreased visibility of the large choroidal vessel layer (Fig 1C).
- Marbled (M) type was defined by slightly dim choroidal reflectivity with obscured vessel margins and dispersion of multiple, amorphous, hyperreflective areas within the layers (Fig 1D).
- Pauci-Vascular (PV) type was defined by a lack of large choroidal vessels with choroidal thickness less than 100 um in the fovea and extrafoveal areas (Fig 1E).

**Fig 2:**
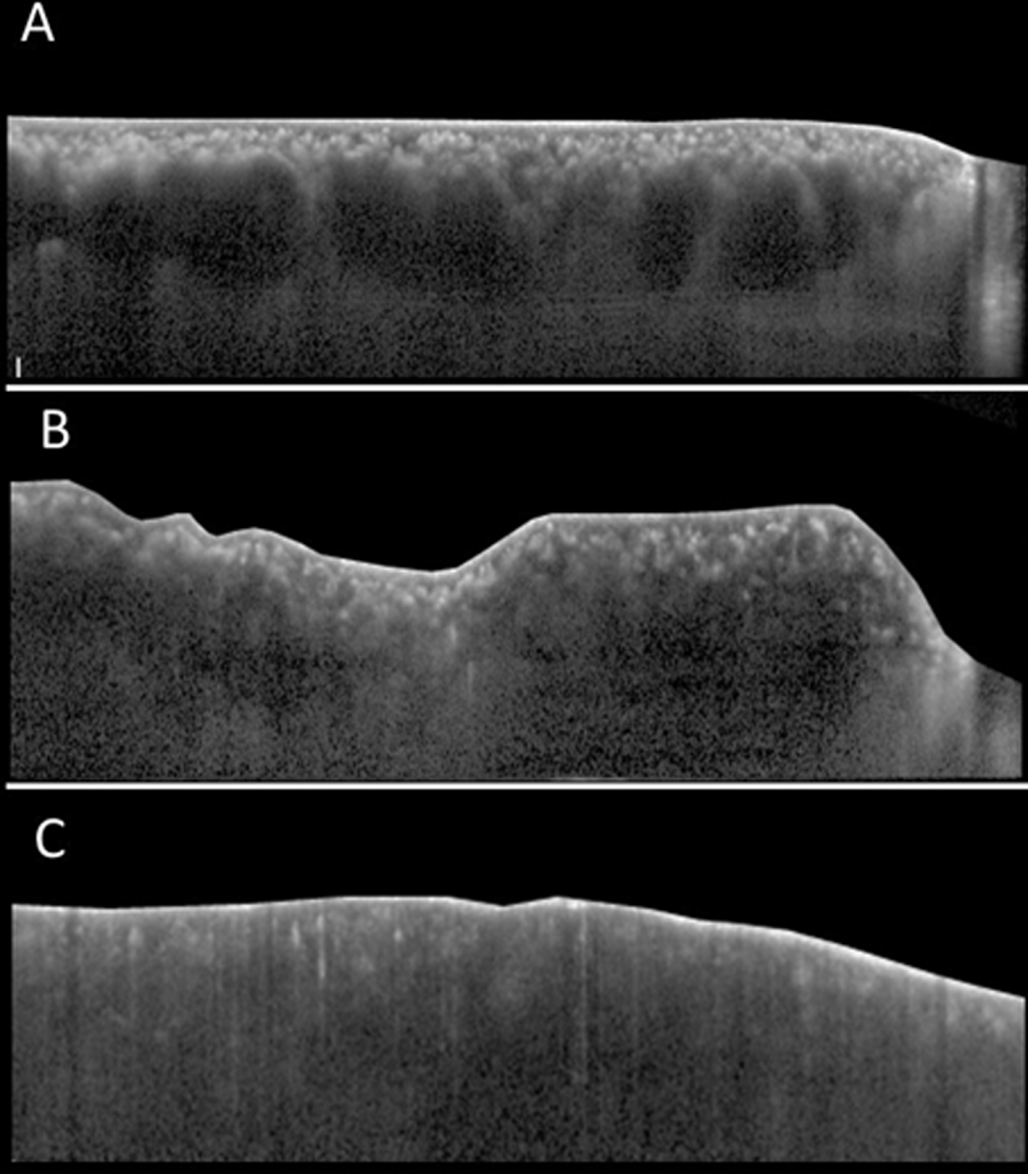
Additional tomographic characteristics of the choroid. Choroidal vessel dilation (Fig 2A): the presence of 3 consecutive vessel lumens with a diameter width of at least 200㎛ in the large choroidal vessel layer is observed. Homogenous hyperreflectivity consistent from the RPE line to the posterior border of the sclera with a constant width of at least 200㎛ is observed (arrows). Convoluted choroid (Fig 2B): the innermost choroidal layer together with the RPE line has a wavy, flexuous appearance. Scleral invisibility (Fig 2C): the chorio-scleral border beneath the choroidal layer is invisible.

Along with morphologic analysis of the choroid, data on the presence of four tomographic characteristics of the choroid was collected. These additional tomographic characteristics were defined as follows (Fig 2):

- Choroidal vessel dilation occurred when three consecutive vessel lumens with a diameter width of at least 200㎛ in the large choroidal vessel layer were detected (Fig 2A).
- Convoluted choroid occurred when the innermost choroidal layer, together with the RPE line, had a wavy, flexuous appearance (Fig 2B).
- Scleral invisibility occurred when the chorio-scleral border beneath the choroidal layer was invisible (Fig 2C).
- Choroidal hyperthickening was defined relative to age. Hyperthickening was defined by subfoveal choroidal thickness over 440 ㎛, 380 ㎛, and 350 ㎛ in subjects in their 40’s, 50’s, and 60’s, respectively. Hyperthinning was defined by thickness under 115 ㎛, 105 ㎛, and 75 ㎛ in subjects in their 40’s, 50’s, and 60’s, respectively. These cut-off values were of two standard deviation from mean subfoveal choroidal thickness of age-matched normal Asian population, which was provided by Fujiwara et al. [16].

Statistical analysis was performed using a commercially available statistical software package (PASW software version 18.0, SPSS). Fisher’s exact test was performed for intergroup comparison of the distribution of five choroidal types (normal vs. each disease), and *P-*values less than 0.017 were considered statistically significant after Bonferroni correction. *P-*values of less than 0.003 and 0.002 after Bonferroni correction were considered statistically significant in the analysis of a type of tomographic classification and a finding of tomographic characteristics when compared to that of normal controls, respectively. The level of intra-observer and inter-observer agreement about choroidal typing was assessed using intra-class correlation coefficients applying a 2-way mixed model with absolute agreement and average measures.

The areas under the ROC (AUROC) curves were acquired for the prediction of CSC, PCV, and VKH.

## Results

The mean age of patients in each disease group was 47.5 (±10.4) years for CSC, 68.2 (±9.2) years for PCV, and 45.1 (±14.7) years for VKH. The normal control group consisted of subjects with a mean age of 57.7(±14.7) years (Table 1).

**Table 1:** Demographic data in each group.

	Normal	CSC	PCV	VKH
Eyes	30	30	30	27
Age (years)	57.7±14.7	47.5±10.4	68.2±9.2	45.1±14.7
Sex ratio (M:F)	0.58 (11:19)	6.50 (26:4)	3.29 (23:7)	2.38 (19:8)

CSC, central serous chorioretinopathy; PCV, polypoidal choroidal vasculopathy; VKH, Vogt-Koyanagi-Harada disease.

### Analysis of the tomographic classification of the choroid

**Fig 3:**
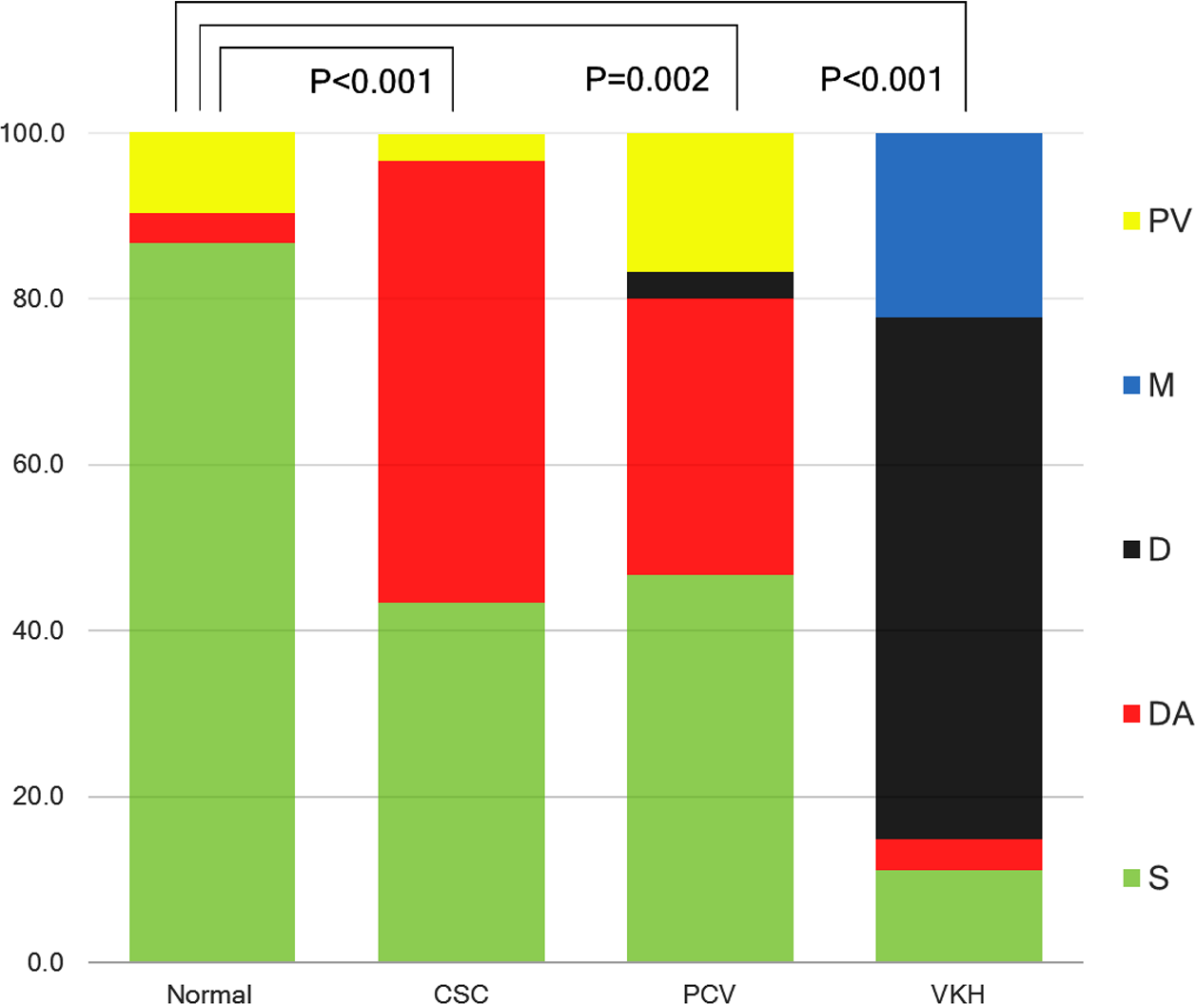
Distribution of choroidal type in each disease. The normal control group consists of either Standard (S) type or Pauci-vascular (PV) type, comprising 96.7% of the total (86.7% and 10.0%, respectively). Over 96% (96.6%) of the central serous chorioretinopathy (CSC) group can be classified into either the Dilated and Attenuated (DA) type or S type (53.3% and 43.3%, respectively). The polypoidal choroidal vasculopathy (PCV) group consisted of the S type (46.7%) and DA type (33.3%) in 80.0% of cases. Vogt-Koyanagi-Harada disease (VKH) showed predominantly Darkened (D) type (63.0%) and Marbled (M) type (22.2%), with these two types comprising 85.2% of the disease group. The distribution of the choroidal type was different in comparison among groups—CSC vs. normal, PCV vs. normal, and VKH vs. normal (*P <* 0.001, *P =* 0.002, and *P <* 0.001, respectively). The *P* value of 0.017 was considered to indicate statistical significance (accounting for a Bonferroni correction).

The majority (96.7%) of the normal control group was classified as either S type or PV type (86.7% and 10.0%, respectively). In contrast, the DA type and S type were prevalent in the CSC group, with a combined frequency of 96.6% (53.3% and 43.3%, respectively). Eighty percent of the PCV group was classified as either S type (46.7%) or DA type (33.3%), and 85.2% of the VKH group was classified as either D type (63.0%) or M type (22.2%). The distribution of choroid type was different between individual disease groups and normal controls: CSC vs. normal (*P <* 0.001), PCV vs. normal (*P =* 0.002), and VKH vs. normal (*P <* 0.001) (Table 2, Fig 3).

**Table 2:** Tomographic classification of the choroid in each disease group.

Group	S type	DA type	D type	M type	PV type	*P-*value*
**Normal** (N = 30)	26 (86.7%)	1 (3.3%)	0 (0%)	0 (0%)	3 (10.0%)	
**CSC** (N = 30)	13 (43.3%)†	16 (53.3%)†	0 (0%)	0 (0%)	1 (3.3%)	<0.001
**PCV** (N = 30)	14 (46.7%)†	10 (33.3%)	1 (3.3%)	0 (0%)	5 (16.7%)	0.002
**VKH** (N = 27)	3 (11.1%)†	1 (3.7%)	17 (63.0%)†	6 (22.2%)	0 (0%)	<0.001

CSC, central serous chorioretinopathy; PCV, polypoidal choroidal vasculopathy; VKH, Vogt-Koyanagi-Harada disease.

* *P-*value indicates intergroup differences in the distribution of tomographic types between normal controls and each disease group. *P-*values less than 0.017 (accounting for a Bonferroni correction) were considered significant.

† Indicates the frequency of tomographic types which have *P-*values less than 0.003 when compared with normal controls using Fischer’s exact test. Accounting for a Bonferroni correction, *P-*values less than 0.003 were considered to indicate statistical significance.

DA type was significantly more frequent in the CSC group than in the control group (53.3% vs. 3.3%, *P <* 0.001). S type was significantly less frequently observed in the CSC group than in the control group (43.3% vs. 86.7%, *P =* 0.001). When comparing PCV with the control group, the DA type had a tendency to appear more frequently (33.3% vs. 3.3%, *P =* 0.006). S type was significantly less frequently observed in the PCV group than in the control group (46.7% vs. 86.7%, *P =* 0.002). There was no difference in the frequency of PV type or D type (3.3% vs. 0.0%, *P =* 1.000) between the two groups (Table 2, Fig 3).

When comparing VKH with the control group, the D type was more prevalent in the VKH group (63.0% vs. 0%, *P <* 0.001), while the S type had a higher prevalence in the normal control group (11.1% vs. 86.7%, *P <* 0.001). The M type tended to be more frequently observed in the VKH group than in the control group (22.2% vs. 0%, *P =* 0.008) (Table 2, Fig 3). Among VKH cases, the acute cases showed high frequency of D type (80.0%), while the recurrent cases showed a high frequency of M type (50.0%) that was significantly higher than that in the control group (*P <* 0.001).

Observing the frequency of the diseases by tomographic type, it was noted that 94.4% of D type and 100% of M type were VKH patients. A total of 92.8% of DA type was either CSC or PCV patients (57.1% in CSC and 35.7% in PCV).

### Analysis of the additional tomographic characteristics of the choroid

In the CSC group, choroidal vascular dilation was observed at a significantly higher frequency (76.7%, *P <* 0.001) than that of the control group (13.3%). Although a higher incidence of choroidal vascular dilation also appeared in the PCV group, it was not a significant difference (36.7% vs. 13.3%, *P =* 0.072) (Table 3).

**Table 3:** Additional tomographic characteristics of the choroid in each disease group.

Group	Vessel Dilation	Convolution	Scleral invisibility	Hyper-thickening	Hyper-thinning
**Normal** (N = 30)	4 (13.3%)	0 (0.0%)	0 (0.0%)	3 (10.0%)	5 (16.7%)
**CSC** (N = 30)	23 (76.7%)*	0 (0.0%)	3 (10.0%)	11 (36.7%)	1 (3.3%)
**PCV** (N = 30)	11 (36.7%)	1 (3.3%)	1 (3.3%)	4 (13.3%)	9 (30.0%)
**VKH** (N = 27)	5 (18.5%)	11 (40.7%)*	19 (70.4%)*	3 (11.1%)	0 (0.0%)

CSC, central serous chorioretinopathy; PCV, polypoidal choroidal vasculopathy; VKH, Vogt-Koyanagi-Harada disease.

* Indicates the frequency of tomographic characteristics which have *P-*values less than 0.003 when compared with normal controls using Fischer’s exact test. Accounting for a Bonferroni correction, *P-*values less than 0.002 were considered to indicate statistical significance.

Convolution was more frequently observed in VKH than in the control group (40.7% vs. 0%, *P <* 0.001) (Table 3). When analyzing the acute stage of VKH, which excluded the recurrent stage, convoluted choroid was observed with a higher prevalence of 66.7% (10/15). Convolution was noted in 50.0% (9/18) of D type, 16.7% (1/6) of M type, and 1.8% (1/56) of S type. It was significantly more frequent in D type than in S type (*P <* 0.001) (Table 4).

**Table 4:** The association between additional tomographic characteristics and the tomographic classification of the choroid.

Choroidal Type	Vessel Dilation	Convolution	Scleral invisibility	Hyper-thickening	Hyper-thinning
**S type** (N = 56)	17 (30.4%)	1 (1.8%)	5 (8.9%)	9 (16.1%)	5 (8.9%)
**DA type** (N = 28)	18 (64.3%)	1 (3.6%)	3 (10.7%)	10 (35.7%)	1 (3.6%)
**D type** (N = 18)	4 (22.2%)	9 (50.0%)*	11 (61.1%)*	2 (11.1%)	0 (0.0%)
**M type** (N = 6)	1 (16.7%)	1 (16.7%)	4 (66.7%)*	0 (0.0%)	0 (0.0%)
**PV type** (N = 9)	3 (33.3%)	0 (0.0%)	0 (0.0%)	0 (0.0%)	9(100.0%)*

* Indicates the frequency of tomographic characteristics which have *P-*values less than 0.002 when compared with S type. Accounting for Bonferroni correction, *P-*values less than 0.002 were considered to indicate statistical significance.

With respect to scleral visibility, the sclera was invisible in 10.0% (3/30) of CSC, 3.3% (1/30) of PCV, 70.4% (19/27) of VKH, and 0% (0/30) of the control group. The VKH group had a significantly higher frequency of invisible sclera when compared to the other groups (*P <* 0.001, respectively) (Table 3). Higher frequency of invisible sclera was observed in the D and M types, as compared to the S type (*P <* 0.001, *P =* 0.002) (Table 4).Choroidal hyperthickening was more frequent in the CSC group as compared to the control group with marginal significance (36.7% vs. 10.0%, *P =* 0.030) (Table 3).

In the comparison between each disease groups, no significant difference was observed between the CSC and PCV groups (*P =* 0.165). However, when compared with the VKH group, the CSC group exhibited significantly higher frequency of DA type and vessel dilation, and lower frequency of D type, convolution and scleral invisibility (all, *P*<0.001). Also, compared with the PCV group, the VKH group exhibited significantly higher frequency of D type, convolution and scleral invisibility. (*P*<0.001) (Table 5). The intra-class correlation coefficients for intra-observer reproducibility showed almost perfect agreement with the level of 0.99 (95%CI: 0.98–1.00, *P*<0.001) and 0.99 (95% CI: 0.98–0.99, *P*<0.001) in the analysis of choroidal tomographic classification and additional tomographic characteristics, respectively. The intra-class correlation coefficients for inter-observer variability also showed fairly good agreement with the level of 0.81 (95% CI: 0.73–0.86, *P*<0.001) and 0.85 (95% CI: 0.82–0.87, *P*<0.001) in the analysis of choroidal tomographic classification and additional tomographic characteristics, respectively.

**Table 5:** Comparison of frequencies of choroidal types and additional tomographic characteristics.

	CSC vs VKH *P-*value	PCV vs VKH *P-*value	CSC vs PCV *P-*value
Difference in overall distribution of choroidal types*	<0.001*	<0.001*	0.165
S type	0.009	0.004	1.000
DA type	<0.001^†^	0.006	0.192
D type	<0.001^†^	<0.001^†^	1.000
M type	0.008	0.008	NA
PV type	1.000	0.053	0.195
Vessel dilation	<0.001^†^	0.151	0.004
Convolution	<0.001^†^	<0.001^†^	1.000
Scleral Invisibility	<0.001^†^	<0.001^†^	0.612
Hyperthickening	0.033	1.000	0.072
Hyperthinning	1.000	0.002	0.012

CSC, central serous chorioretinopathy; PCV, polypoidal choroidal vasculopathy; VKH, Vogt-Koyanagi-Harada disease.

* Accounting for Bonferroni correction, *P*-values less than 0.008 were considered significant.

^†^ As for the inter-group comparison of the distribution of choroid types or tomographic characteristcs, *P*-values less than 0.0017 were considered significant.

Table 6 shows the AUROC for prediction of CSC, PCV, and VKH using the representative choroidal classification and characteristics. DA type was a strong predictive factor for CSC (AUROC = 0.698, *P =* 0.001). D type (AUROC = 0.809, *P <* 0.001), choroidal convolution (AUROC = 0.698, *P =* 0.002), and scleral invisibility (AUROC = 0.830, *P <* 0.001) were also strong predictive values for VKH. The combination of D type, M type, choroidal convolution, and scleral invisibility was the strongest predictive factor for VKH (AUROC = 0.972, *P <* 0.001).

**Table 6:** The diagnostic value of tomographic classification and characteristics in central serous chorioretinopathy, polypoidal choroidal vasculopathy, and Vogt-Koyanagi-Harada disease.

Tomographic Classification and Characteristics	AUROC (95% CI)		
	CSC	PCV	VKH
DA type	0.698* (0.579–0.816)	0.563 (0.441–0.686)	NA
D type	NA	NA	0.809** (0.694–0.925)
M type	NA	NA	0.611 (0.478–0.744)
D type + M type	NA	NA	0.920** (0.839–1.000)
Convolution	NA	NA	0.698* (0.568–0.828)
Scleral Invisibility	NA	NA	0.830** (0.722–0.937)
D type + M type + Convolution + Scleral Invisibility	NA	NA	0.972** (0.943–1.000)

CSC, central serous chorioretinopathy; PCV, polypoidal choroidal vasculopathy; VKH, Vogt-Koyanagi-Harada disease.

NA: not applicable

* *P-*value<0.01

** *P-*value<0.001.

## Discussion

**Fig 4:**
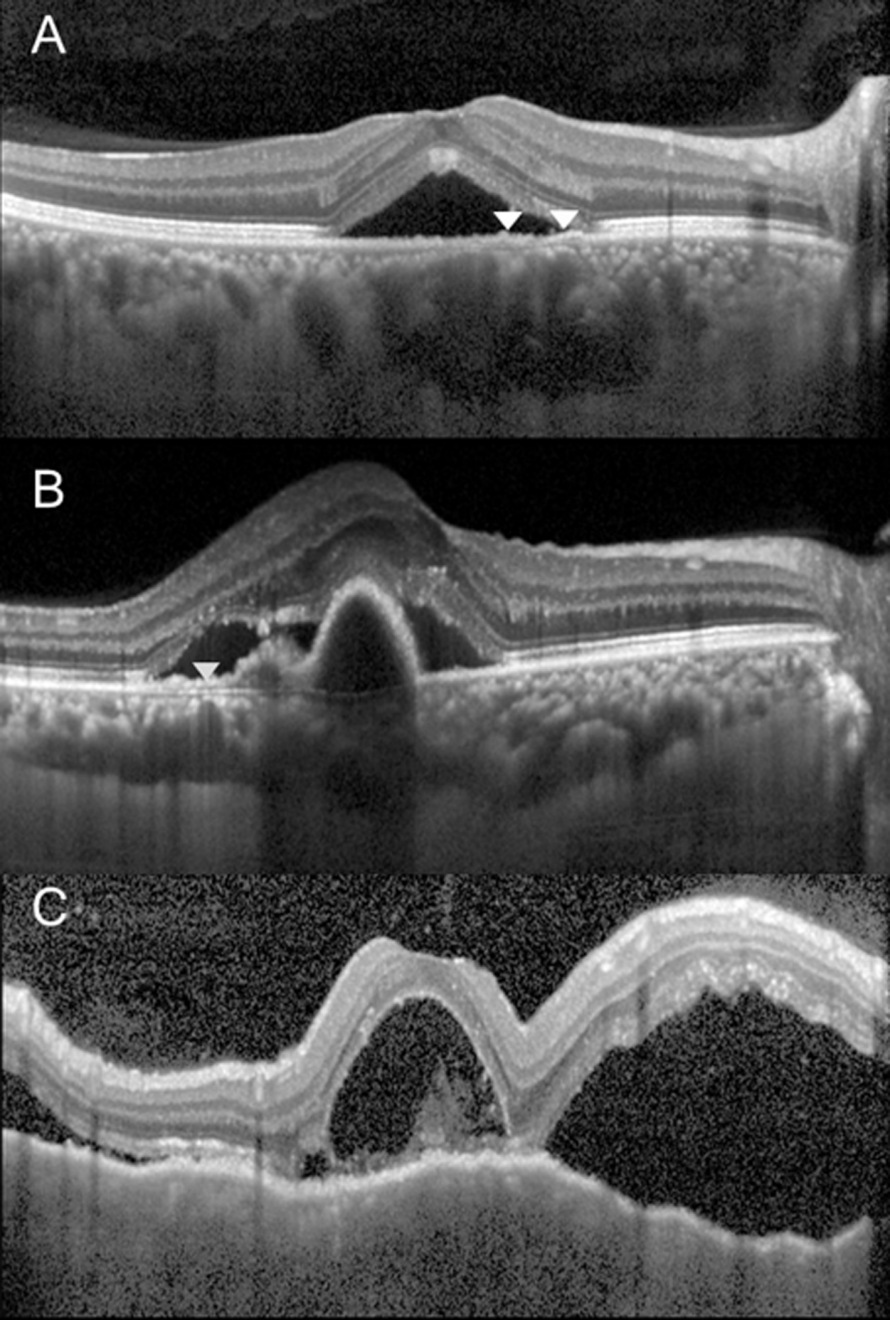
Optical coherence tomography of the patients with central serous chorioretinopathy (CSC), polypoidal choroidal vasculopathy (PCV), and Vogt-Koyanagi-Harada disease (VKH). The choroidal morphologic changes in the respective diseases are represented. In 49 year-old male with CSC (Fig 4A), neurosensory retinal detachment with subretinal fluid is observed. The choroid is characterized by Dilated and Attenuated (DA) type (arrowheads), choroidal vascular dilation, and choroidal hyperthickening. PCV (Fig 4B), 61 year-old female, subretinal fluid with PED are well depicted. The choroid is classified as a DA type (arrowheads). VKH (Fig 4C), 38 year-old female, multifocal neurosensory detachments along with the convoluted choroid and invisible sclera. The choroid appears to be a typical Darkened (D) type.

Significant differences in the choroidal morphology between major choroidal thickening diseases were appreciated by our classification system of choroid. The choroidal morphologic features on OCT were reported in diverse conditions such as diabetic retinopathy [18], PCV [19], VKH [20, 21], CSC [22], focal choroidal excavation [23] and normal population [24]. However, as far as we are aware, this is the first report to develop a novel morphologic classification system of the choroid and apply it to the major pachychoroidopathies. The representative choroidal OCT findings of CSC, based on those significantly different from normal controls, are the DA type and choroidal vascular dilation. The representative choroidal OCT findings of PCV were DA type. The representative OCT findings in VKH were high frequencies of D type or M type, convolutions, and scleral invisibility. Fig 4 demonstrates representative tomographic appearances of the choroid in each disease.

A major finding of this study was that the DA type was more frequently found in CSC and PCV compared with normal controls. Over 90% of the cases classified as DA type were CSC or PCV. In particular, the high frequency of the DA type in CSC may be related to the pathogenesis of CSC. Choroidal hyperpermeability is known to be a cause of CSC, inducing secondary mechanical disruption of the RPE, accumulation of subretinal fluid, or appearance of pigment epithelial detachment, etc. [13, 25–29]. Delayed filling and arterial non-perfusion reported to appear concomitantly in areas with late choroidal hyperpermeability [14] may serve as theoretical evidence to explain the tomography of the DA type, which showed focal areas of attenuation in innermost layer appearing concurrently with large dilated choroidal vessels. Also, a study on the histologic findings of PCV reported hyalinization and obstruction of inner choroidal vessels below the RPE, and abnormal dilation of the outer large choroidal vessels [30]. Such histologic changes may be related to high prevalence of the DA type in PCV.

Another important finding was that when D type and M type were observed, the cases were almost always found to be VKH. The two choroidal types showed diagnostic specificity for VKH. They also had a high sensitivity, as 85.2% of the 27 patients with VKH could be classified into either one of the two types. D type was apparently noted more frequently in acute phase of VKH than in convalescent phase. It is possibly due to thickened choroid related to acute choroiditis, although the determination of D type was based on significantly decreased visibility of the large choroidal vessel layer. VKH is a non-necrotizing, diffuse, granulomatous inflammation involving the uvea. In the acute uveitic stage, there are diffuse infiltrating lymphocytes and macrophages located close to the uveal melanocytes in the choroid, and multinucleated giant cells and epitheloid cells [31]. Proteinaceous fluid exudates produced in this stage accumulate in the choroid stroma and may obscure the choroidal vessels margin explaining the diffuse, homogenous, hypo-reflective form classified as the D type. The M type is consistent with findings from the convalescent phase of VKH. Six patients defined as M type showed sunset glow fundus, which is characteristic of the convalescent stage of VKH. Histologically, loss of melanin pigment occurs from the damage of choroidal melanocytes in this stage, and focal aggregations of lymphocytes are observed [31]. In OCT images of the M type, choroidal layers appear sparse and dim, and multiple, amorphous, hyperreflective areas are located within the layers of the stroma. We speculate that the M type is due to increased reflectance of the choroid from the loss of melanocytes and persistent damage to the retinal pigment epithelium during the convalescent stage, and increased proportion of extravascular space arising from vascular attenuation or choroidal fibrosis after chronic choroiditis.

The PV type, defined as thinning of the choroid and decrease in large choroidal vessel layers, could be considered normal variation of the choroid rather than being associated with disease, as it also occasionally appears in the control group with no statistically significant difference compared to disease groups. This type could also be a manifestation of age-related choroidal atrophy as presented by Spaide [32]. VKH can be classified as retinal detachment type and disc swelling only type [33]. VKH cases in the current study were limited to those in the active phase of the retinal detachment type.

In this study, additional tomographic characteristics of the choroid were also analyzed. The frequencies of choroidal vessel dilation and age-relative hyperthickening were significantly higher in the CSC group, consistent with previous studies [14, 27, 29, 34, 35]. However, choroidal vascular dilation on tomography was arbitrarily defined, as there were no previous definitions, and the adequacy of such criteria may need to be further explored.

Folding in the choroid and RPE found in VKH has been reported in various studies [20,36–38]. Kato et al., reported that 71.4% of subjects with the disease showed retinal folds [20]. In our study, when limited to the acute stage of VKH, retinal folds were observed in 66.7%, which is consistent with other reports [21]. VKH also had a high prevalence of invisible sclera. This is thought to be attributed to obscuration of the chorio-scleral junction line from increased choroidal infiltration.

A notable finding of this study is that the distribution of choroidal type in CSC and PCV show no statistically meaningful difference. From this, it is possible to speculate that the two diseases share a common choroidal pathology. Choroidal hyperpermeability and punctate hyperfluorescent spots have been reported in both diseases [14, 29, 39, 40]. A secondary PCV case resulting from CSC has also been reported, and other reports imply that CSC is a risk factor for PCV [39, 41–43]. The observations in current study support the possible role of CSC in the pathogenesis of PCV.

Another meaningful finding of this study is that such tomographic classification and choroidal characteristics may aid in the diagnosis of the aforementioned diseases due to relatively high diagnostic predictive values. When using the combination of D type, M type, convolution and scleral invisibility as a predictive factor for VKH, the AUROC value is 0.972, which implies strong reliability and usefulness as a diagnostic tool. We also believe that DA type for CSC can serve as good predictive factors, with AUROC value 0.698 (Table 6).

In this study, representative pachychoroidopathies with high incidence in Asians were selected and a classification system was applied. The clinical relevance of the results can be summarized as follows. First, analysis of choroid thickness and morphology is important for disease diagnosis. Although angiographic studies are indispensable in diagnosing these diseases, angiographic imaging alone can be quite obscure in a significant proportion of these patients. For instance, although it is often difficult to distinguish PCV from other subtypes of exudative AMD, if the case presents with a thickened choroid, then the diagnosis of typical exudative AMD or retinal angiomatous proliferation can be ruled out [10, 44]. Distinguishing between atypical CSC and VKH is another example that may sometimes pose a diagnostic challenge despite adequate angiographic imaging. Although both diseases accompany choroidal thickening, current study indicates that choroidal tomographic morphology is significantly different. That is, identification of DA type choroid in ambiguous cases strongly favors the diagnosis of atypical CSC. In contrast, D type, M type, and choroidal convolution were specific markers for VKH. Thus, tomographic morphologic information of the choroid as provided by the current study may greatly enhance diagnostic specificity in these diseases. Second, most prior studies regarding morphological changes in the choroid have focused on a single disease entity. In contrast, the present study highlighted the differences as well as similarities between them by comparing morphologic features of the choroid between the major pachychoroidopathies. For example, this study revealed that CSC and PCV share a similar choroidal tomographic morphology. The result may significantly support the hypothesis of so-called CSC-pachychoroid pigment epitheliopathy-PCV axis [43, 45]. Thus, studying choroidal morphology may also provide pathogenic implications in a certain group of diseases.

There are several limitations in this study. First, because only three diseases with choroidal thickening were studied, all possible classifications of the choroid were not included. Other pachychoroidal diseases such as a variety of inflammatory diseases may also show choroidal morphologic alterations. Further study on this issue would be meaningful. Second, this study also compared the active phase of each disease with normal eyes, irrespective of whether the disease was an initial attack or a recurrent case. Therefore, it is a cross-sectional study of the active phase of each disease. Longitudinal observations of the diseases may lead to differences in tomographic classification or in the characteristics of the choroid. Third, due to the retrospective nature of this study, we designated normal fellow eyes with enhanced depth imaging OCT from unilateral epiretinal membrane patients as the control group. Epiretinal membrane is the leading vitreo-retinal interface disorder, and there is no evidence that the choroid in fellow eyes of epiretinal membrane patients should be considered different from the normal population. However, we believe that including normal eyes from healthy controls with no ocular diseases would eliminate potential selection bias. Despite such limitations, this study stands out because morphologic patterns in the thickened choroid were classified and characterized, and their correlation with certain diseases was addressed. We believe that such efforts will help enhance understanding of the pathophysiology of these diseases and lead to the development of less invasive diagnostic and monitoring techniques for these diseases.

In conclusion, a new choroidal morphologic classification system utilizing OCT may provide useful tool with discrimination ability among choroidal thickening diseases. CSC and PCV are characterized by focal loss of the inner choriocapillaris layer just above the dilated large choroidal vessels. In addition, choroidal vascular dilation and marked thickening were more prevalent in the eyes with CSC. On the other hand, along with convolution and scleral invisibility, choroidal darkening due to diffuse and homogenous hypo-reflectivity on OCT is highly specific finding for the diagnosis of VKH.

## Supporting Information

S1 Appendix Dataset of major choroidal thickening diseases and tomographic features. (DOCX)
