# Supplementary material for: Morphologic Characteristics of Choroid in the Major Choroidal Thickening Diseases, Studied by Optical Coherence Tomography
Source: PLoS One. 2016 Jan 14;11(1):e0147139. doi: 10.1371/journal.pone.0147139 (PMC4713229; doi:10.1371/journal.pone.0147139)
Supplement: S1 Appendix — (DOCX) [file pone.0147139.s001.docx]

S1 appendix. Dataset of major choroidal thickening diseases and tomographic features.

| Case | Disease | Type | Hyper-thickening | Hyper-thinning | VKH  stage | Vessel  Dilation | Convolution | Scleral instability |
| --- | --- | --- | --- | --- | --- | --- | --- | --- |
| 1 | csc | da | 1 | 0 |  | 1 | 0 | 0 |
| 2 | csc | da | 0 | 0 |  | 1 | 0 | 0 |
| 3 | csc | da | 0 | 0 |  | 0 | 0 | 0 |
| 4 | csc | da | 1 | 0 |  | 1 | 0 | 0 |
| 5 | csc | s | 0 | 0 |  | 0 | 0 | 0 |
| 6 | csc | da | 1 | 0 |  | 1 | 0 | 1 |
| 7 | csc | s | 1 | 0 |  | 1 | 0 | 0 |
| 8 | csc | s | 0 | 0 |  | 1 | 0 | 0 |
| 9 | csc | s | 0 | 0 |  | 0 | 0 | 1 |
| 10 | csc | s | 0 | 0 |  | 1 | 0 | 0 |
| 11 | csc | s | 1 | 0 |  | 1 | 0 | 0 |
| 12 | csc | s | 0 | 0 |  | 1 | 0 | 0 |
| 13 | csc | s | 1 | 0 |  | 1 | 0 | 0 |
| 14 | csc | da | 0 | 0 |  | 1 | 0 | 0 |
| 15 | csc | da | 1 | 0 |  | 1 | 0 | 0 |
| 16 | csc | da | 0 | 0 |  | 1 | 0 | 0 |
| 17 | csc | pv | 0 | 1 |  | 0 | 0 | 0 |
| 18 | csc | da | 1 | 0 |  | 1 | 0 | 0 |
| 19 | csc | s | 0 | 0 |  | 0 | 0 | 0 |
| 20 | csc | da | 1 | 0 |  | 1 | 0 | 0 |
| 21 | csc | s | 0 | 0 |  | 1 | 0 | 1 |
| 22 | csc | da | 0 | 0 |  | 1 | 0 | 0 |
| 23 | csc | da | 1 | 0 |  | 1 | 0 | 0 |
| 24 | csc | s | 0 | 0 |  | 1 | 0 | 0 |
| 25 | csc | da | 0 | 0 |  | 0 | 0 | 0 |
| 26 | csc | da | 0 | 0 |  | 1 | 0 | 0 |
| 27 | csc | s | 0 | 0 |  | 1 | 0 | 0 |
| 28 | csc | s | 0 | 0 |  | 0 | 0 | 0 |
| 29 | csc | da | 1 | 0 |  | 1 | 0 | 0 |
| 30 | csc | da | 0 | 0 |  | 1 | 0 | 0 |
| 31 | pcv | da | 0 | 0 |  | 0 | 0 | 0 |
| 32 | pcv | da | 1 | 0 |  | 1 | 0 | 0 |
| 33 | pcv | s | 0 | 0 |  | 0 | 0 | 0 |
| 34 | pcv | d | 0 | 0 |  | 1 | 0 | 0 |
| 35 | pcv | da | 0 | 1 |  | 1 | 0 | 0 |
| 36 | pcv | s | 0 | 0 |  | 0 | 0 | 0 |
| 37 | pcv | da | 0 | 0 |  | 1 | 0 | 0 |
| 38 | pcv | s | 0 | 1 |  | 0 | 0 | 0 |
| 39 | pcv | s | 0 | 1 |  | 1 | 0 | 0 |
| 40 | pcv | s | 0 | 0 |  | 0 | 0 | 0 |
| 41 | pcv | da | 0 | 0 |  | 0 | 0 | 0 |
| 42 | pcv | pv | 0 | 1 |  | 0 | 0 | 0 |
| 43 | pcv | s | 0 | 1 |  | 0 | 0 | 0 |
| 44 | pcv | s | 1 | 0 |  | 0 | 0 | 0 |
| 45 | pcv | s | 0 | 0 |  | 0 | 0 | 0 |
| 46 | pcv | s | 0 | 0 |  | 0 | 0 | 0 |
| 47 | pcv | s | 0 | 0 |  | 1 | 0 | 0 |
| 48 | pcv | s | 0 | 0 |  | 0 | 0 | 0 |
| 49 | pcv | pv | 0 | 1 |  | 1 | 0 | 0 |
| 50 | pcv | da | 0 | 0 |  | 0 | 1 | 1 |
| 51 | pcv | pv | 0 | 1 |  | 1 | 0 | 0 |
| 52 | pcv | pv | 0 | 1 |  | 1 | 0 | 0 |
| 53 | pcv | pv | 0 | 1 |  | 0 | 0 | 0 |
| 54 | pcv | s | 0 | 0 |  | 0 | 0 | 0 |
| 55 | pcv | da | 1 | 0 |  | 1 | 0 | 0 |
| 56 | pcv | s | 0 | 0 |  | 0 | 0 | 0 |
| 57 | pcv | da | 0 | 0 |  | 0 | 0 | 0 |
| 58 | pcv | da | 0 | 0 |  | 0 | 0 | 0 |
| 59 | pcv | da | 0 | 0 |  | 0 | 0 | 0 |
| 60 | pcv | s | 1 | 0 |  | 1 | 0 | 0 |
| 61 | vkh | m | 0 | 0 | convalescent | 0 | 0 | 1 |
| 62 | vkh | d | 0 | 0 | acute | 1 | 1 | 1 |
| 63 | vkh | d | 0 | 0 | acute | 0 | 1 | 1 |
| 64 | vkh | d | 0 | 0 | convalescent | 1 | 0 | 1 |
| 65 | vkh | d | 0 | 0 | acute | 0 | 1 | 1 |
| 66 | vkh | d | 1 | 0 | convalescent | 0 | 0 | 0 |
| 67 | vkh | m | 0 | 0 | convalescent | 0 | 0 | 0 |
| 68 | vkh | m | 0 | 0 | convalescent | 0 | 0 | 1 |
| 69 | vkh | m | 0 | 0 | convalescent | 0 | 1 | 1 |
| 70 | vkh | da | 0 | 0 | acute | 0 | 0 | 1 |
| 71 | vkh | m | 0 | 0 | convalescent | 1 | 0 | 0 |
| 72 | vkh | d | 0 | 0 | acute | 0 | 1 | 1 |
| 73 | vkh | d | 1 | 0 | convalescent | 0 | 0 | 0 |
| 74 | vkh | d | 0 | 0 | convalescent | 0 | 0 | 0 |
| 75 | vkh | d | 0 | 0 | acute | 0 | 1 | 1 |
| 76 | vkh | d | 0 | 0 | acute | 0 | 1 | 1 |
| 77 | vkh | d | 0 | 0 | convalescent | 0 | 0 | 0 |
| 78 | vkh | d | 0 | 0 | acute | 0 | 0 | 0 |
| 79 | vkh | s | 0 | 0 | convalescent | 0 | 0 | 1 |
| 80 | vkh | m | 0 | 0 | convalescent | 0 | 0 | 1 |
| 81 | vkh | d | 0 | 0 | acute | 1 | 1 | 1 |
| 82 | vkh | s | 1 | 0 | acute | 0 | 1 | 1 |
| 83 | vkh | d | 0 | 0 | acute | 0 | 1 | 1 |
| 84 | vkh | d | 0 | 0 | acute | 0 | 1 | 1 |
| 85 | vkh | d | 0 | 0 | acute | 0 | 0 | 1 |
| 86 | vkh | d | 0 | 0 | acute | 0 | 0 | 0 |
| 87 | vkh | s | 0 | 0 | acute | 1 | 0 | 1 |
| 88 | normal | s | 0 | 0 |  | 0 | 0 | 0 |
| 89 | normal | s | 0 | 0 |  | 0 | 0 | 0 |
| 90 | normal | s | 0 | 0 |  | 0 | 0 | 0 |
| 91 | normal | s | 0 | 0 |  | 0 | 0 | 0 |
| 92 | normal | s | 0 | 0 |  | 0 | 0 | 0 |
| 93 | normal | pv | 0 | 1 |  | 0 | 0 | 0 |
| 94 | normal | pv | 0 | 1 |  | 0 | 0 | 0 |
| 95 | normal | da | 0 | 0 |  | 0 | 0 | 0 |
| 96 | normal | s | 0 | 0 |  | 0 | 0 | 0 |
| 97 | normal | pv | 0 | 1 |  | 0 | 0 | 0 |
| 98 | normal | s | 0 | 0 |  | 0 | 0 | 0 |
| 99 | normal | s | 0 | 1 |  | 0 | 0 | 0 |
| 100 | normal | s | 0 | 1 |  | 0 | 0 | 0 |
| 101 | normal | s | 0 | 0 |  | 0 | 0 | 0 |
| 102 | normal | s | 0 | 0 |  | 0 | 0 | 0 |
| 103 | normal | s | 0 | 0 |  | 0 | 0 | 0 |
| 104 | normal | s | 0 | 0 |  | 0 | 0 | 0 |
| 105 | normal | s | 0 | 0 |  | 1 | 0 | 0 |
| 106 | normal | s | 0 | 0 |  | 0 | 0 | 0 |
| 107 | normal | s | 0 | 0 |  | 0 | 0 | 0 |
| 108 | normal | s | 1 | 0 |  | 0 | 0 | 0 |
| 109 | normal | s | 1 | 0 |  | 0 | 0 | 0 |
| 110 | normal | s | 0 | 0 |  | 0 | 0 | 0 |
| 111 | normal | s | 0 | 0 |  | 1 | 0 | 0 |
| 112 | normal | s | 0 | 0 |  | 1 | 0 | 0 |
| 113 | normal | s | 0 | 0 |  | 1 | 0 | 0 |
| 114 | normal | s | 0 | 0 |  | 0 | 0 | 0 |
| 115 | normal | s | 0 | 0 |  | 0 | 0 | 0 |
| 116 | normal | s | 1 | 0 |  | 0 | 0 | 0 |
| 117 | normal | s | 0 | 0 |  | 0 | 0 | 0 |
